# Syntonic phototherapy versus part time occlusion for treatment of refractive amblyopia

**DOI:** 10.1038/s41598-025-90283-x

**Published:** 2025-02-27

**Authors:** Sanaa Ahmed Mohamed, Adel Mohammed Abdel-Wahab Khalil, Maha Emhammed Almokhtar Aljaghmani

**Affiliations:** 1 Department of Ophthalmology, Faculty of Medicine for Girls, Al-Azhar University, New Damietta, Egypt; 2 Department of Ophthalmology, Faculty of Medicine for Girls, Al-Azhar University, Cairo, Egypt

**Keywords:** Amblyopia, Anisometropia, Syntonic phototherapy, Part-time occlusion, Health care, Optics and photonics

## Abstract

To evaluate the effectiveness of syntonic phototherapy and compare it with partial time occlusion to improve visual acuity in cases of refractive amblyopia. This study is a prospective, comparative, and randomized study. It included 40 patients. Their mean age *±* SD was 14.45 ± 10.03 years (Range: 6–45 years). Twenty patients were subjected to partial time occlusion of the sound eye, and 20 received syntonic phototherapy treatment.The study revealed that there was statistically significant improvement in visual functions, UCVA, BCVA, AOP, and functional visual field in patients who were subjected to syntonic phototherapy, whereas the improvement of UCVA and BCVA in patients who were subjected to conventional treatment was satisfactory but less than that reported by syntonic therapy. Visual acuity increased significantly in patients with amblyopia after syntonic phototherapy as compared to partial occlusion therapy.

## Introduction

Amblyopia, a neurodevelopmental condition caused by abnormal visual experience in childhood, reduces best-corrected visual acuity unilaterally or bilaterally and has a prevalence of 2–4% ^1^. In industrialized nations, amblyopia is the leading cause of impaired vision in both children and adults, and it has far-reaching societal and economic consequences^1^. Amblyopia is considered a cortical visual loss where two eyes first interact. Without treatment in the first 8–10 years of life, it might cause lifelong visual impairment. While adults can improve with treatment, early detection, and treatment yield the best results^2^.

Common risk factors for amblyopia include refractive error, namely anisometropic hyperopia, strabismus, which interferes with binocular fusion, and, less commonly, an obstruction in the optical pathway that diminishes retinal picture quality, such as congenital cataract. The impaired vision is not caused by any obvious eye or visual pathway problems but rather by some diseases that interfere with the normal development of the visual system after birth^3^.

Uncorrected refractive errors are widely regarded as the primary factor leading to amblyopia. Refractive amblyopia can be classified into two primary categories. Anisometropic amblyopia is a condition where one eye has a different refractive defect than the other, resulting in unilateral amblyopia. Isoametropic amblyopia is a condition in which both eyes have amblyopia because of a large and similar refractive defect^4^.

Amblyopia treatment aims to improve the eye’s connection to the brain and block sound eye impulses. The treatment reduces picture blur, which hinders cortical connection formation. It also reduces bilateral inhibition to equalize eye dominance in the ocular dominance columns. During the early stages of life, this strategy is most effective. Amblyopia has a variety of proposed treatments. It has been found in one modality that the amblyopic eye can improve its vision gain when the normal eye is not stimulated visually. Restoring monocular function is usually accomplished by covering the amblyopic eye with a patch from the healthy eye^5–7^.

Traditionally, it was common to cover the stronger eye throughout the child’s waking hours for several days equal to their age and then cover the weaker eye for one day. This provides the benefit of binocular interaction between both eyes and eliminates the possibility of occlusion amblyopia. Part-time occlusion (PTO) is now widely accepted as the preferred method for treating amblyopia in Western countries. However, the patient’s compliance with this method is very low globally, so the need for other treatment modalities is critical^8,9^.

Light, sound, and movement are all examples of energy-based stimuli that have been demonstrated to increase brain activity and neuroplasticity^10^. As a result, these stimulants may have promise for enhancing brain function by reactivating dormant neuronal circuits or by constructing new cortical networks^11^. Light can activate certain neuronal groupings. Light frequencies carry energy and deliver information. Colors excite enzymes and control cell activities and chemical production. Different wavelengths of light may affect the organism differently^12,13^.

Visible light, perceptible to the human eye, is a continuum of wavelength electromagnetic radiation ranging from 400 to 700 nm. The hue of light is governed by its specific wavelength^14,15^. Colour perception is facilitated by the absorption of light through three types of cone cells in the eye, namely blue, green, and red, with corresponding wavelengths of 400–450 nm, 500–570 nm, and 610–750 nm, respectively^16^.

Syntonic is a therapeutic approach that utilizes non-coherent, non-polarized, wide-spectrum light to stimulate the primary visual cortex via the retina directly. This makes it effective for addressing visual dysfunctions such as strabismus, amblyopia, brain injuries, learning difficulties, and certain ocular pathologies. In the field of optometry, synthetic phototherapy has been applied practically and clinically to patients with strabismus and amblyopia. However, there is a lack of scientific research on its specific effects on these patients’ brain and visual systems.

A red filter selectively absorbs all wavelengths of light except for red. As a result, red light specifically stimulates the cones in the retina, particularly in the fovea, where cone cells are most abundant. The red light increases the accumulation of electrical charge in the cell membrane, which then improves the charge of nerve cells to overcome synaptic resistance and reduce amblyopia^17^.

Monochromatic filters have been used to treat light sensitivity in patients with traumatic brain injuries, change the visual-evoked potential (VEPs) in children diagnosed with visual stress, improve perception of patients with symptoms of visual processing disorder, and reduce cortical hyper-activation in patients who suffer from migraine, suggesting that the neural activity of these patients, may be altered by wavelength-dependent processes. Taking into consideration the cortical origin of these conditions and the possibility of modulating brain activity using different wavelengths, the same principle could be applied to patients with strabismus and amblyopia^18^. Light stimulation and phototherapy, which employs a combination of two or more wavelengths, can modulate the Alpha-wave, interhemispheric connections, anteroposterior gradient, and brain coherence^10^ and functional connectivity patterns on brain networks measured with fMRI, are light-dependent^19^.

Alpha omega pupil (pupillary stress) test: is characterized by the abnormal re-dilatation of the pupil during direct constant light stimulation. The speed and amount of this dilation give information on how dominant the sympathetic system is over the parasympathetic. In a normal pupillary reaction, this pupil would constrict and take an average of 15 s to dilate again^20^.

Functional color field testing or campimetry: Optometric phototherapy providers use kinetic perimetry to assess patient responses to four targets (green, red, blue, and white) during diagnosis and treatment. Kinetic color perimetry is performed throughout treatment to identify whether to modify or stop phototherapy^21^.

This work aims to evaluate the effectiveness of syntonic phototherapy and compare syntonic phototherapy with partial time occlusion to improve visual acuity in cases of refractive amblyopia.

## Patients and methods

This study is a prospective, comparative, and randomized study. It was done between December 2020 and April 2022. It was held at Al Zahraa University Hospital. It included 40 patients aged from 3 to 50 years. Patients were classified into two groups:

### Group I

Included 20 patients who were subjected to occlusion therapy.

### Group II

Included 20 patients subjected to syntonic phototherapy.

Patients in each group were subdivided into three age groups to assess the effect of age on the treatment results: Younger children (3–7 years old), older children (8–18 years old), and adult patients (> 18 years old).

### Ethical consideration

This study was approved by the institutional review board of the Faculty of Medicine for Girls, Al-Azhar University, Cairo, and according to the declaration of Helsinki reg (RHBIRB201812201). The patients’ parents gave their written agreement to use their information in the study and the research. All parents of the patients signed the informed consent to publish their information and images in an online open-access publication. The nature of the study was explained to each patient before starting treatment, including its purpose, the procedure, the benefits, the expected duration, the potential risks involved, any discomfort that may be caused, and possible complications. Each patient was informed that participation is voluntary and that he/she may withdraw from the study at any time without giving a reason. Patients were invited to participate in the study if they met the inclusion criteria and did not have exclusion criteria.

### The inclusion criteria

Refractive errors predisposing to amblyopia, an interocular best-corrected visual acuity difference of at least 2 lines of acuity between the two eyes without a history of patching or atropine penalization, use of spectacles or contact lenses for at least 3 months before they join the study.

### The Exclusion Criteria

There is an ocular disease that could contribute to decreased visual acuity, the presence of strabismus, pregnancy or lactating women, and patients with a history of seizures.

### Pre-treatment assessments

After taking both verbal and written consent, all patients were subjected to a detailed history and complete ophthalmological examination, including Estimation of Uncorrected (UCVA) and best-corrected visual acuities (BCVA) using a Snellen chart; results were converted to logMAR values for analysis, cycloplegic Autorefractometry, slit-lamp biomicroscopy, and indirect ophthalmoscopy.

**Patients of group II were additionally subjected to** The alpha-omega pupil test, functional visual field test, and pulse rate before and after the first session.

Amblyopia treatment was started with a full spectacle correction according to the cycloplegic refraction. Patients wore their prescribed spectacles for at least 3 months and were followed at the ophthalmology Outpatient clinic monthly. Occlusion of the sound eye with patching or syntonic phototherapy was prescribed if there was no vision improvement in the amblyopic eye. The success of amblyopia treatment was based on post-treatment BCVA 0.1 (LogMAR) or better.

**Group I**: included 20 patients in total: eight males (40%) and 12 females (60%). The age range was from 6 to 37 years (mean ± SD was 10.70 ± 6.81). Of the twenty patients, five patients (25%) had left-eye amblyopia, seven patients (35%) had right-eye amblyopia, and eight patients (40%) had bilateral amblyopia.

Patients were subjected to partial time occlusion therapy, and the patching strategy was based on different visual acuities^22^. Patients were followed every 2 weeks for 3 months and were subjected to an estimation of UCVA and BCVA for each eye using a Snellen chart at each follow-up visit. During patching hours, children were commonly advised to perform far and near activities or activities requiring hand-eye coordination, such as reading, drawing, playing with blocks, playing video games, tracing pictures, or completing puzzles. They were advised to do saccadic and pursuits exercises by using longitudinal saccadic charts and swinging ball pursuits.

**Fig. 1:**
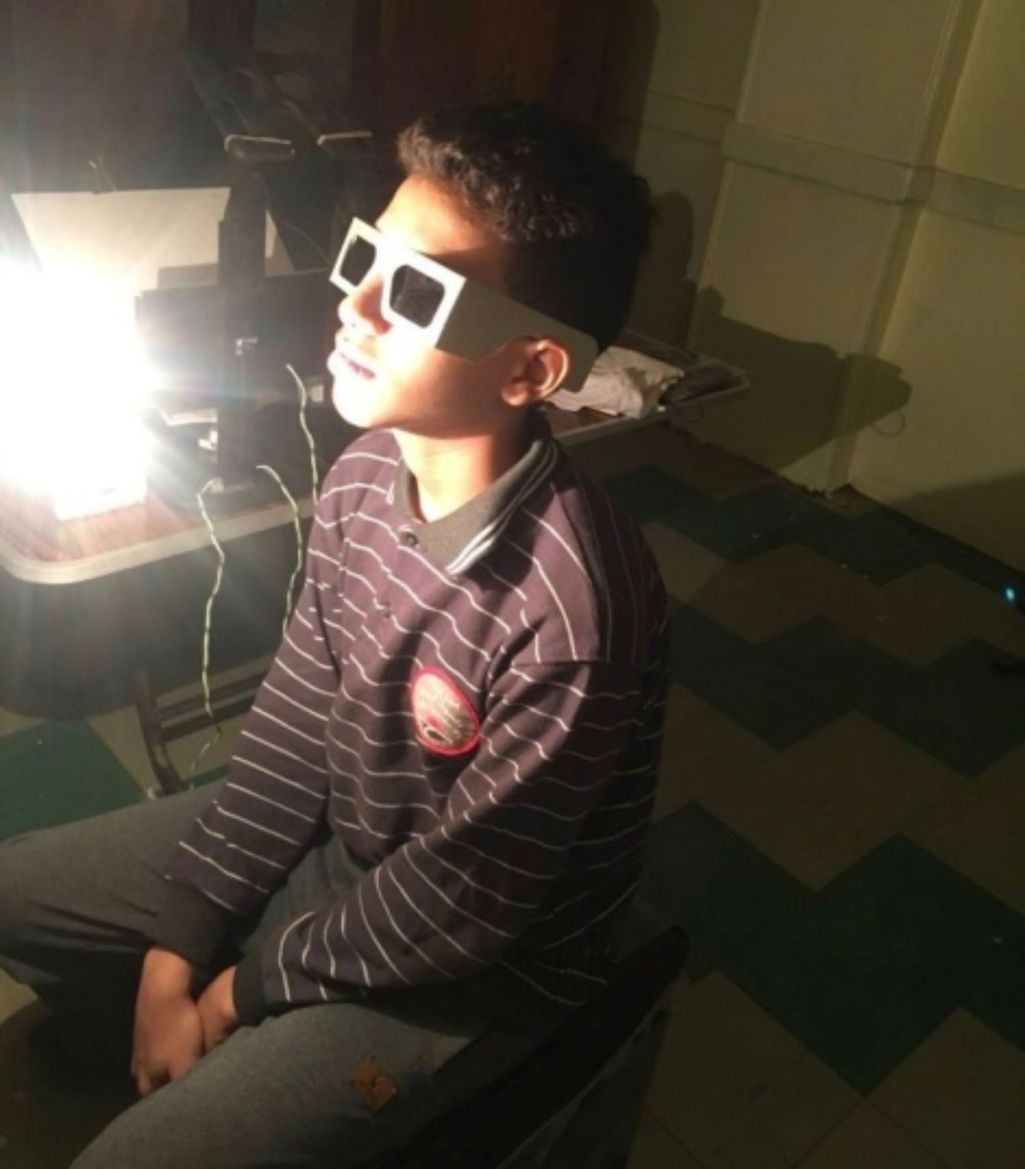
A patient under syntonic treatment.

**Group II**: included 20 patients in total, Ten males (50%) and ten females (50%), with a mean age of 18.20 ± 11.41. The age range was from 7 to 45 years. Of the twenty patients, Seven patients (35%) had left eye amblyopia, four patients (20%) had right eye amblyopia, and nine patients (45%) had bilateral amblyopia. Patients were subjected to syntonic phototherapy. The plan for each patient was to receive 30 sessions of syntonic phototherapy. Still, because the resolution of amblyopia was achieved with fewer sessions, 1 of the 20 patients received 8 sessions, 3 received 16 sessions, 8 received 24 sessions, and 8 received 30 sessions. Syntonic phototherapy was performed in each session by wearing two syntonic filters: Upsilon Omega for 2 min and Theta for 5 min. This treatment was repeated four times a week in a completely dark room. Patients were asked to fixate at a light source (12 V 50 W Halogen bulb) at a distance of 20 inches while wearing their filter glasses. The patient was kept in a comfortable position throughout the sessions and was allowed to move his / her eye if fatigued. The patient was exposed to light binocularly in the case of bilateral amblyopia, but only the amblyopic eye was exposed to light in the case of unilateral amblyopia (Fig. 1).

Patients were followed every 2 weeks (every 8 sessions) for 3 months. In each follow-up visit, sis patients were subjected to an estimation of UCVA and BCVA for each eye. The pulse rate was assessed before and following the first session to monitor the syntonic phototherapy’s vascular interaction via its impact on the autonomic nervous system. The alpha-omega pupil test was performed as follows: in a scotopic room, the patient looked binocularly at a stimulus placed 6 m away, with one of the pupils illuminated with a flashlight about 8 cm away. The pupillary reaction to light was observed and graded into one of 5 grades. When the pupil remains miotic for at least 10 s, it is considered a normal response; dilating before this time is considered an abnormal response.

### The alpha omega pupil (αω) was graded as follows

Grade 4 = No pupil response. Grade 3 = Pupil constricts, then dilates and stays dilated. Grade 2 = Pupil constricts then dilates – pupil fluctuates between constriction and dilation. Grade 1 = Pupil constricts, then dilates and re-constricts. Grade 0 = Pupil constricts and remains constricted.

**Fig. 2:**
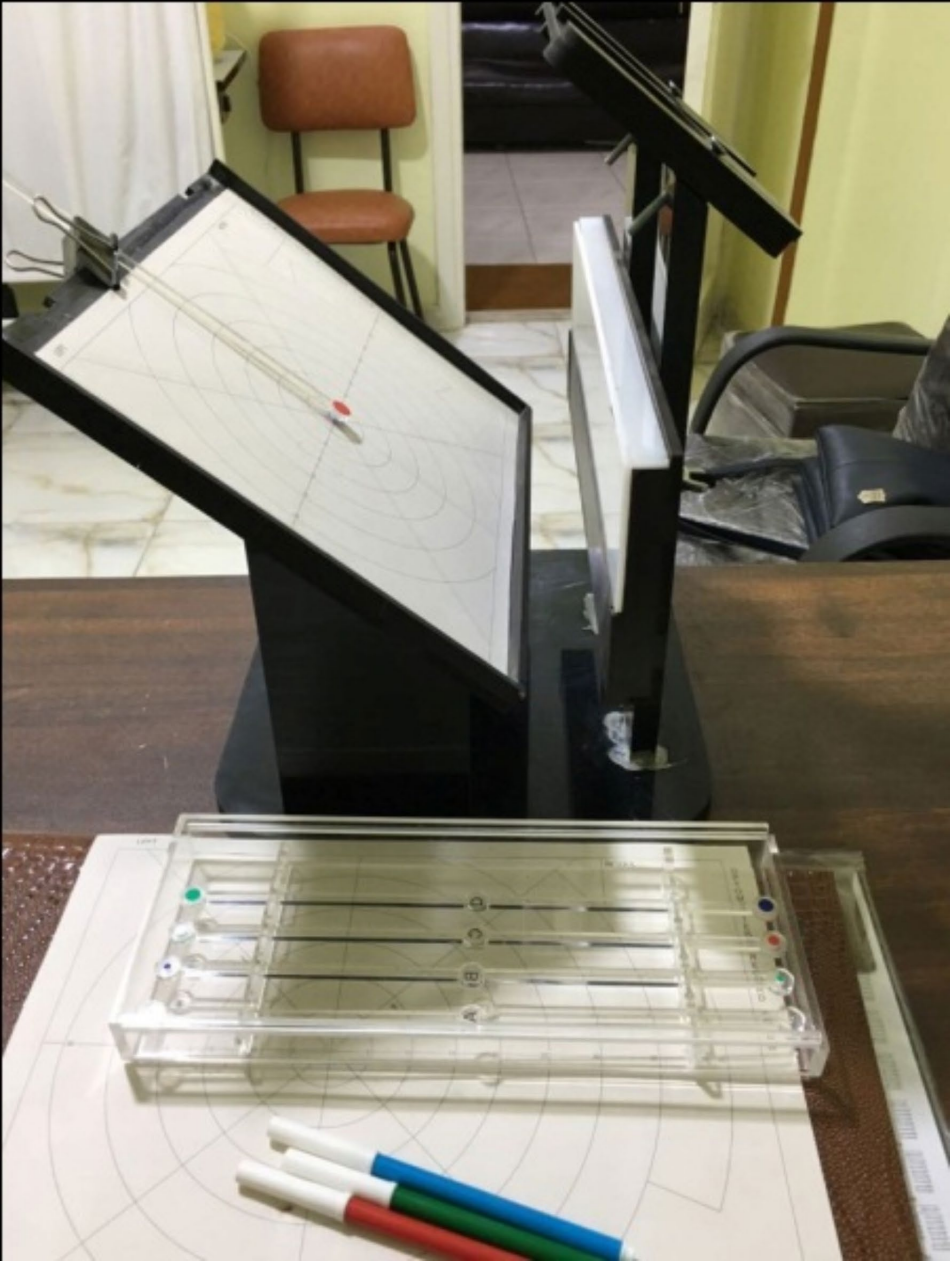
Visual field being plotted by kinetic campimeter.

The functional color field test was performed by campimeter at each follow-up visit to assess the treatment’s response via field expansion (Fig. 2).

**Fig. 3:**
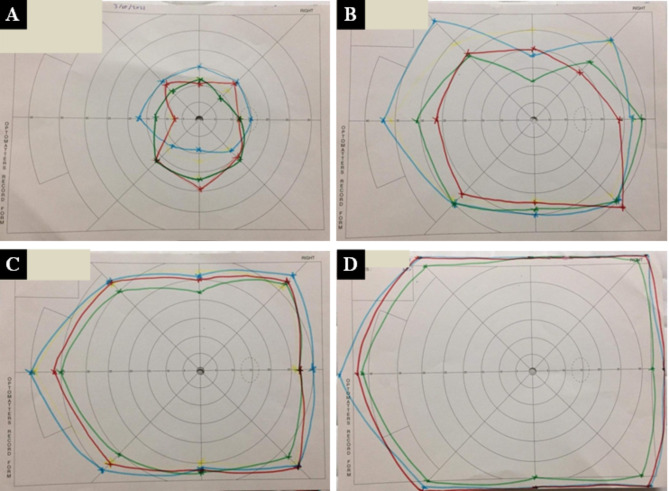
(A) shows the pre-syntonic visual field, (B) shows the visual field after 8 sessions, (C) shows the visual field after 16 sessions, (D) shows the visual field after 24 sessions.

The kinetic functional color field was plotted by campimeter as follows: In the case of bilateral amblyopia, the right eye was measured first, followed by the left eye, unless the amblyopic eye was tested only. Without wearing their prescribed spectacles, participants placed their eyes on the viewing apparatus and were instructed to maintain looking at the fixation target at the center of the chart. With 3 mm green, red, and then blue targets in this order from non-seeing to seeing area, we measured 8 cardinal meridians (0, 45, 90, 135, 180, 225, 270, and315) (Fig. 3). We placed a fixation target in the center of the chart corresponding to the color of the tested field target. We tried to use a consistent, steady speed of movement. The participants were briefed that each target would be approached from the periphery (i.e., non-seeing to seeing) and that they would have to verbalize the target color (green, red, or blue) once they could confidently identify it. We made measurement marks on the second sheet, not on the sheet the patient is looking at. Patients adjusted their prescribed glasses to obtain the best corrected visual acuity, which changed as their amblyopia improved.

### USED ​​FILTERS

Two different filters were used to carry out this portion of the study. In each session, the patient was treated with syntonic phototherapy for 7 min in a room with mesopic conditions. Filters used were the following:

#### Upsilon Omega (υω)

It combines two filters, Upsilon + Omega, and its name refers to the color indigo.

#### Theta

The color corresponding to this name is yellow (Green with red).

Following a session of syntonic phototherapy, vision therapy activities lasted 30 min. The principle of our training program is based on the publication of Morya, et al.^23^. Vision therapy includes orthoptic vision therapy and behavior/perceptual vision therapy. Orthoptic training was divided into three categories: accommodative, anti-suppression, and vergence therapy, *w*hich varied from monocular-to-monocular fixation in a binocular field (MFBF) to biocular and binocular activities.

Marsden ball, red-green playing cards, longitudinal saccadic chart, and eye-hand coordination activities were used for behavior instruction. The program enhances pursuit, saccade, and binocular function. Patients did all vision therapy exercises without glasses. Instead of using the same equipment, the half-hour vision therapy patients received treatments that followed the same principles and were prescribed based on their performance. We partitioned the half-hour program into two parts. During the initial 15 min, the patients were trained using specialized equipment designed to address the issue of antisuppression. This included the use of antisuppression charts, workbooks, and the Hart Chart. During the previous fifteen minutes, behavior training was administered to the patients, as the tools employed were more captivating and amusing, which could potentially alleviate any fatigue resulting from the training.

### Statistical analysis

The data was analyzed in version 20.0 of SPSS (Chicago, Illinois, USA). Quantitative data were presented as mean ± SD. Qualitative data were presented as frequency and percentage. Paired sample t-test of significance was used to compare the related sample. Pearson’s correlation coefficient (r) test assessed the degree of association between two sets of variables.

**Table 1:** Comparison between group I and group II regarding demographic data.

		Group I	Group II	Test value	*P*-value	Sig.
		No. = 20	No. = 20			
Age (years)	Mean ± SD	10.70 ± 6.81	18.20 ± 11.41	−2.578•	0.010	S
	Range	6–37	7–45			
	3–7 yrs	7 (35.0%)	3 (15.0%)	8.800*	0.012	S
	8–18 yrs	12 (60.0%)	8 (40.0%)			
	> 18 yrs	1 (5.0%)	9 (45.0%)			
Sex	Male	8 (40.0%)	10 (50.0%)	0.404*	0.525	NS
	Female	12 (60.0%)	10 (50.0%)			
Laterality	Unilateral	12 (60.0%)	11 (55.0%)	0.102*	0.749	NS
	Bilateral	8 (40.0%)	9 (45.0%)			
Anterior segment examination	Normal	20 (100.0%)	20 (100.0%)	NA	NA	NA
Fundus picture	Normal	17 (85.0%)	18 (90%)	0.239*	0.625	NS
	Tigroid fundus	3 (15.0%)	2 (10%)			
**Cycloplegic refraction**						
Sphere (D)	Mean ± SD	−1.05 ± 5.56	−1.10 ± 4.51	−0.122≠	0.903	NS
	Range	−12–7	−12–7			
Cylinder (D)	Mean ± SD	−1.66 ± 1.40	−0.94 ± 3.04	−0.194≠	0.846	NS
	Range	−5–0.5	−6.75–7			
Axis (°)	Mean ± SD	83.60 ± 75.59	119.97 ± 63.29	−1.162≠	0.245	NS
	Range	0–180	4–180			

P-value > 0.05: Non significant; P-value ≤ 0.05: Significant; P-value ≤ 0.01: Highly significant.

*: Chi-square test; •: Independent t-test; ≠: Mann-Whitney test.

## Results

A total number of 40 patients were included in our study. There were non-statistically significant differences between group I and group II regarding sex, side, and laterality, while there was a statistically significant difference between both groups regarding the mean age of the studied patients (*p* < 0.05) (Table 1). There was a statistically significant difference between both studied groups as regards the number of improved lines (BCVA) (*p* < 0.05) (Table 2).

**Table 2:** Comparison between group I and group II regarding no. of lines improvement (BCVA).

No. of improved lines (BCVA)	Group I	GroupII	Test value	P-value	Sig.
	No. = 20	No. = 29			
Mean ± SD	2.65 ± 1.84	4.34 ± 2.77	−2.490≠	0.013	S
Range	0–6	2–13			

≠: Mann-Whitney test.

Table 3 shows highly significant differences between both groups of patients with age ≥ 8 years as regards UCVA and BCVA at visits 2, 3, and 4 (*p* < 0.05). Table 4 shows that there were highly statistically significant improvements in UCVA and BCVA at different times during the treatment duration among study group I (*p* < 0.001). Table 5 shows highly statistically significant improvements in AOP, UCVA, and BCVA at different follow-up visits in group II (*p* < 0.001).

**Table 3:** Comparison between group I and group II in patients aged 8 years or more.

		Group I	Group II	Test value	*P*-value	Sig.
		MeanSD	MeanSD			
Pre treatment						
UCVA	Mean ± SD	0.82 ± 0.32	0.78 ± 0.40	0.247≠	0.807	NS
	Range	0.4–1.3	0.2–1.3			
BCVA	Mean ± SD	0.54 ± 0.27	0.54 ± 0.32	−0.025≠	0.981	NS
	Range	0.2–1.1	0.2–1.3			
**Visit 1**						
UCVA	Mean ± SD	0.82 ± 0.32	0.54 ± 0.38	2.118≠	0.043	S
	Range	0.4–1.3	0.1–1.3			
BCVA	Mean ± SD	0.54 ± 0.27	0.36 ± 0.31	1.682≠	0.104	NS
	Range	0.2–1.1	0–1.3			
**Visit 2**						
UCVA	Mean ± SD	0.77 ± 0.34	0.34 ± 0.27	3.785≠	0.001	HS
	Range	0.4–1.3	0–0.9			
BCVA	Mean ± SD	0.49 ± 0.27	0.23 ± 0.20	3.003≠	0.006	HS
	Range	0.1–1	0–0.7			
**Visit 3**						
UCVA	Mean ± SD	0.72 ± 0.28	0.21 ± 0.20	5.449≠	0.000	HS
	Range	0.4–1.1	0–0.6			
BCVA	Mean ± SD	0.45 ± 0.23	0.11 ± 0.14	4.620≠	0.000	HS
	Range	0.1–1	0–0.4			
**Visit 4**						
UCVA	Mean ± SD	0.67 ± 0.28	0.26 ± 0.14	3.684≠	0.002	HS
	Range	0.4–1.1	0.1–0.4			
BCVA	Mean ± SD	0.40 ± 0.20	0.14 ± 0.13	3.011≠	0.007	HS
	Range	0.1–0.8	0–0.4			

P-value > 0.05: Non significant; P-value ≤ 0.05: Significant; P-value ≤ 0.01: Highly significant.

≠: Mann-Whitney test.

Table 6 shows that there were statistically insignificant differences between different age groups (3–7 years vs.8–18 years vs. > 18 years) as regards UCVA, BCVA, and AOP among patients of group II (*p* > 0.05). Table 7 shows that after 30 sessions, the mean percentage values of blue field expansion increased from 18.86 ± 5.18 to 78.10 ± 39.69, green field expansion increased from 19.93 ± 6.01 to 69.55 ± 42.92, and red field expansion increased from 18.10 ± 4.86 to 83.88 ± 45.40.

**Table 4:** UCVA and BCVA at different follow-up visits in patients of group I.

Group I		Pre-treatment		Visit 1		Visit 2		Visit 3		Visit 4		Visit 5		Visit 6		Test value		*P*-value		Sig.	
UCVA	Mean ± SD	0.82 ± 0.33		0.82 ± 0.34		0.75 ± 0.32		0.67 ± 0.27		0.62 ± 0.26		0.59 ± 0.27		0.55 ± 0.26		25.779≠		< 0.001		HS	
	Range	0.2–1.3		0.2–1.3		0.2–1.3		0.2–1.1		0.2–1.1		0.2–1.1		0.2–1.1							
BCVA	Mean ± SD	0.56 ± 0.28		0.54 ± 0.27		0.47 ± 0.24		0.41 ± 0.21		0.36 ± 0.20		0.33 ± 0.18		0.29 ± 0.16		28.588≠		< 0.001		HS	
	Range	0.2–1.1		0.2–1.1		0.1–1		0.1–1		0.1–0.8		0.1–0.8		0–0.6							

P-value > 0.05: Non significant; P-value ≤ 0.05: Significant; P-value ≤ 0.01: Highly significant.

≠: Friedman test.

Table 8 shows that the patients who achieved a success rate in group I was 25% of the total (5 out of 20 patients), while in group II, the success rate was 86.2% (BCVA ≤ 0.1 Log-Mar) (mean number of sessions needed was 17.6 ± 8 sessions).

**Table 5:** AOP, UCVA, and BCVA among group II at different follow-up visits.

Group II		Pre-treatment	Visit 1	Visit 2	Visit 3	Visit 4	Test value	*P*-value	Sig.
UCVA	Mean ± SD	0.73 ± 0.36	0.49 ± 0.31	0.30 ± 0.23	0.20 ± 0.20	0.26 ± 0.14	33.851≠	< 0.001	HS
	Range	0.2–1.3	0.1–1.3	0–0.9	0–0.6	0.1–0.4			
BCVA	Mean ± SD	0.49 ± 0.30	0.31 ± 0.26	0.20 ± 0.19	0.12 ± 0.16	0.15 ± 0.14	18.726≠	< 0.001	HS
	Range	0.2–1.3	0 − 1.3	0–0.7	0–0.5	0–0.4			
AOP	Mean ± SD	2.00 ± 0.92	2.00 ± 0.92	1.65 ± 0.99	1.24 ± 0.90	1.88 ± 0.35	10.111≠	0.001	HS
	Range	0–3	0–3	0–3	0–3	1–2			

P-value > 0.05: Non significant; P-value ≤ 0.05: Significant; P-value ≤ 0.01: Highly significant.

≠: Friedman test.

**Table 6:** Effect of age on UCVA, BCVA, and AOP in patients of group II.

		3–7 yrs	8–18 yrs	> 18 yrs	Test value	*P*-value	Sig.
		No. = 3	No. = 8	No. = 9			
Pre-treatment							
UCVA	Mean ± SD	0.80 ± 0.35	0.84 ± 0.35	0.73 ± 0.44	0.147≠	0.864	NS
	Range	0.4–1	0.3–1.3	0.2–1.3			
BCVA	Mean ± SD	0.50 ± 0.44	0.59 ± 0.39	0.50 ± 0.27	0.154≠	0.858	NS
	Range	0.2–1	0.2–1.3	0.2–1			
**Visit 1**							
UCVA	Mean ± SD	0.40 ± 0.10	0.51 ± 0.36	0.57 ± 0.41	0.237≠	0.792	NS
	Range	0.3–0.5	0.1–1.3	0.1–1.1			
BCVA	Mean ± SD	0.17 ± 0.15	0.39 ± 0.39	0.33 ± 0.23	0.590≠	0.565	NS
	Range	0–0.3	0.1–1.3	0–0.7			
AOP	Mean ± SD	2.33 ± 0.58	1.88 ± 0.64	2.00 ± 1.23	0.251≠	0.781	NS
	Range	2–3	1–3	0–3			
**Visit 2**							
UCVA	Mean ± SD	0.15 ± 0.07	0.29 ± 0.22	0.40 ± 0.32	0.851≠	0.446	NS
	Range	0.1–0.2	0.1–0.7	0–0.9			
BCVA	Mean ± SD	0.15 ± 0.07	0.21 ± 0.22	0.25 ± 0.19	0.227≠	0.800	NS
	Range	0.1–0.2	0–0.7	0–0.5			
AOP	Mean ± SD	1.67 ± 0.58	1.50 ± 0.76	1.78 ± 1.30	0.153≠	0.859	NS
	Range	1–2	1–3	0–3			
**Visit 3**							
UCVA	Mean ± SD	0.00 ± 0.00	0.13 ± 0.14	0.29 ± 0.23	2.483≠	0.122	NS
	Range	0–0	0–0.4	0–0.6			
BCVA	Mean ± SD	0.00 ± 0.00	0.04 ± 0.08	0.19 ± 0.16	3.300≠	0.069	NS
	Range	0–0	0–0.2	0–0.4			
AOP	Mean ± SD	1.00 ± 0.00	1.00 ± 0.82	1.50 ± 1.07	0.618≠	0.553	NS
	Range	1–1	0–2	0–3			
**Visit 4**							
UCVA	Mean ± SD	-	0.15 ± 0.07	0.30 ± 0.14	1.891≠	0.228	NS
	Range	-	0.1–0.2	0.1–0.4			
BCVA	Mean ± SD	-	0.05 ± 0.07	0.18 ± 0.13	1.654≠	0.255	NS
	Range	-	0–0.1	0.1–0.4			
AOP	Mean ± SD	-	2.00 ± 0.00	1.83 ± 0.41	0.300≠	0.604	NS
	Range	-	2–2	1–2			

P-value > 0.05: Non significant; P-value ≤ 0.05: Significant; P-value ≤ 0.01: Highly significant.

≠: Kruskal-Wallis test.

## Discussion

Our aim in this study was to evaluate syntonic phototherapy’s effectiveness and compare it with partial time occlusion to improve visual acuity in cases of amblyopia.

**Table 7:** Effect of syntonic phototherapy on color visual field.

		Total no. = 29
Pre		
Blue	Mean ± SD	18.86 ± 5.18
	Range	14–31
Green	Mean ± SD	19.93 ± 6.01
	Range	13–34
Red	Mean ± SD	18.10 ± 4.86
	Range	12–29
**% of change at last visit**		
Blue	Mean ± SD	78.10 ± 39.69
	Range	3.23–128.57
Green	Mean ± SD	69.55 ± 42.92
	Range	0–146.15
Red	Mean ± SD	83.88 ± 45.40
	Range	10.34–166.67

The clinical findings from the current study suggest that, when necessary, syntonic phototherapy can actively change the visual process, fostering new visual abilities while improving old ones. No prior research in the literature has documented the impact of these syntonic phototherapy filters, intended for strabismus and amblyopia patients, on neural activity. However, quantitative electroencephalography studies have shown that light stimulation and phototherapy using a combination of two or more of these wavelengths can modulate alpha waves, interhemispheric connections, anteroposterior gradient, and brain coherence^10^. Likewise, a pilot study on healthy participants reported by Argilés, et al.^19^ showed that functional connectivity patterns on brain networks measured with fMRI are light-dependent. Therefore, the cortical origin of amblyopia should be taken into consideration. We expect to see the effect of these monochromatic filters on the visual pathway, which connects the eyes to the cortex and modulates neural activity.

**Table 8:** Success rate in patients of group I and group II.

	Group I patients who achieved BCVA 0.1 LogMAR or better
No	15 (75.0%)
Yes (after 3 months)	5 (25.0%)
	**Group II who achieved BCVA 0.1 LogMAR or better (no. of eyes = 29)**
No	4 (13.8%)
Yes	25 (86.2%)
*8 session*	*7 (28.0%)*
*16 session*	*9 (36.0%)*
*24 session*	*6 (24.0%)*
*30 session*	*3 (12.0%)*
*Mean ± SD*	*17.6 ± 8*
*Range*	*8–32*

We found that refractive amblyopia treatment using Syntonic phototherapy resulted in a better improvement in UCVA as well as BCVA than that with part-time occlusion at different follow-up visits with statistically significant differences, especially in patients with age > 8 years. This finding is consistent with a previous study reported by Chen, et al.^24^, who showed that the phototherapy group was better than the patching group as regards the visual function of children with amblyopia, as well as it shortened the recovery time of the visual functions in children with amblyopia.

Among the syntonic phototherapy patients’ group, we observed that, of the 29 amblyopic eyes, 9 patients had bilateral amblyopia, and 11 patients had unilateral amblyopia. Twenty-five amblyopic eyes (86.2%) were cured, whereas 4 eyes (13.8%) attained satisfactory improvement regardless of the severity of amblyopia, as both eyes were subjected to light at the same time. The success of amblyopia treatment (86.2%) was based on post-treatment BCVA 0.1 (LogMAR) or better. We found the same thing as Ivandic and Ivandic^25^, who found that 90% of adult eyes with amblyopia showed a statistically significant improvement in best-corrected distant visual acuity following phototherapy. According to their reports, some of their eyes even achieved 6/6 visual acuity. There seemed to be a correlation between baseline visual acuity and the extent of improvement.

We found a highly significant improvement in both UCVA and BCVA among age groups > 18, and there are insignificant differences between the improvement of amblyopia and the age groups (3-7yrs,8–18 yrs, and > 18 yrs). We also confirmed that there are statistically significant improvements in UCVA and BCVA at different follow-up visits among the syntonic phototherapy group. In our study, the mean value of BCVA before syntonic therapy was 0.49 ± 0.30 (LogMAR). After syntonic therapy, it improved to 0.15 ± 0.14 (LogMAR). In the study of Younus, et al.^17^, amblyopes had an average visual acuity of 0.62 ± 0.33 before syntonic therapy and 0.40 ± 0.36 after the procedure. According to their research, amblyopes’ visual acuity was significantly different before and after the syntonic phototherapy (*p* = 0.00).

Our study found a highly significant improvement in visual acuity after a mean number of sessions of 17.6 ± 8 sessions. This result agrees with a study reported by Younus, et al.^17^, who found that only 20 sessions were enough to show significant improvements in visual acuity and contrast sensitivity. They reported a high compliance rate among patients subjected to syntonic therapy. Red filter glasses made the kids joyful, and syntonic therapy was easy and effective for their parents.

In this study, patients of the syntonic group were followed for three months after treatment cessation. During the follow-up visits, we found an insignificant difference between the last session’s visual acuity reading and that at the last follow-up visit, three months after cessation of the treatment. This finding is in line with the study reported by^10^; they found a highly significant improvement in the light therapy group regarding visual acuity 4 months after cessation of the treatment.

We found a highly significant expansion in the functional fields of red, green, and blue colors among the syntonic phototherapy group. There are few reviews about the efficacy of the syntonic phototherapy treatment on the functional color field outcomes. In this study, we compared the functional color field at the different follow-up visits during treatment and after its cessation for 3 months.

Our study also focused on the AOP, which showed a highly statistically significant improvement at different follow-up visits in the syntonic group.

In our study, we observed the pulse rate before and after the first session in the syntonic phototherapy group. Theoretically, syntonic phototherapy has a vascular interaction through its effect on the autonomic nervous system, which may lead to an increase or decrease in the pulse rate after exposure to light. However, we did not find robust changes in pulse rate before and after light exposure. We attributed that to our study’s short time exposure to light, which did not exceed 7 min in every session.

We found a statistically significant improvement in UCVA and BCVA at different follow-up visits among patients of group I. This finding is consistent with a previous study reported by Zakaria Eid, et al.^26^, who assessed BCVA changes after part-time occlusion in children with amblyopia. They found a statistically significant improvement in BCVA after amblyopia treatment.

Our results showed insignificant statistical differences between the two age groups (3–7 yrs vs. 8–18 yrs) regarding UCVA and BCVA among the studied group I. This finding agrees with a previous study reported by Jia, et al.^27^, who found no statistically significant change in the success rate of treatment of anisometropic amblyopia in patients older than 12 years of age.

The findings of Park, et al.^28^ contrast our results. They reported a study to evaluate the efficacy of 6-hour part-time occlusion treatment combined with near activities in unilateral amblyopia. Of the 47 patients younger than 7 years old, 43 (or 91%) had positive outcomes. Seven individuals (or 64%) out of eleven who were seven years old or older had positive outcomes. There was a notable disparity in the success rate between the two groups when looking at age. Differences in amblyopia severity and etiology were not statistically significant by gender. We don’t have any explanation for this result except that the age range of the group aged above 7 years was 8–15 years, with only one patient aged 37 years, who showed no improvement in his visual acuity at all but didn’t give robust effect on the statistical results. At least two factors may contribute to the diminished efficacy of amblyopia medication in older children, according to research by Chen, et al.^29^. Contrary to popular belief, the central nervous system’s flexibility does not diminish with age in children. New evidence from studies treating amblyopia suggests that this plasticity persists into puberty. Second, older children may be less compliant when treated.

Among the patients in group I, 25% (5 out of 20) were cured, while 75% (15 out of 20) saw satisfactory improvement in their condition. These individuals achieved the success rate of amblyopia treatment, as determined by a post-treatment BCVA of 0.1 (LogMAR) or better. The current conclusion contradicts the study conducted by Lee and Lee^30^, which aimed to assess the effectiveness of part-time occlusion therapy for anisometropic amblyopia in children aged eight years or older. In their study, Lee and Lee^30^ observed that the average pre-treatment best-corrected visual acuity (BCVA) score, together with its standard deviation (SD), was 0.51 ± 0.23 (LogMAR). The average post-treatment best-corrected visual acuity (BCVA) was 0.03 ± 0.01 (LogMAR), and the success rate, defined as achieving a post-treatment BCVA of 0.1 (LogMAR) or better, was 96.43%. The post-treatment best corrected visual acuity (BCVA) was obtained within an average of 4.79 ± 3.35 months after treatment.

This study compares 2 h and 6 h of patching time. Our finding showed more improvement in 6-hour occlusion therapy. This finding agrees with a study reported by Singh, et al.^31^, who showed significant visual acuity improvement 18 weeks after the occlusion therapy. They reported that six-hour occlusion treatment was significantly more effective than two-hour occlusion.

Different study populations, qualifying criteria, types of amblyopia, measuring devices, and co-existing variables such as refractive defects likely account for the discrepancies in these publications’ findings.

To the best of our knowledge, this is the first study on amblyopia to examine the efficacy of syntonic phototherapy in enhancing visual acuity, color visual field, and AOP. It’s the first of its kind in Egypt to evaluate the efficacy of syntonic phototherapy vs. part-time occlusion in treating refractive amblyopia.

Our study is limited by its relatively modest sample size. Our study has a shorter duration compared to the duration stated in previous studies. However, the current study indicates that part-time occlusion therapy and syntonic therapy have a positive impact. It is advisable to do more extensive research with a longer duration of follow-up to investigate the problems of relapse and the degree of improvement achieved with part-time occlusion therapy.

## Conclusion

In many cases, amblyopia treatment is successful even after age 8–10 years if the predisposing cause of amblyopia is treated and amblyopia therapy is performed. Syntonic phototherapy is an effective treatment modality for refractive amblyopia. Syntonic phototherapy is superior to part-time occlusion in the treatment of refractive amblyopia.

## Data Availability

The datasets used and/or analyzed during the current study available from the corresponding author on reasonable request.
